# Occurrence of Thromboembolic Events and Mortality Among Hospitalized Coronavirus 2019 Patients: Large Observational Cohort Study of Electronic Health Records

**DOI:** 10.1055/a-1937-9692

**Published:** 2022-11-18

**Authors:** Alex C. Spyropoulos, James M. Crawford, Yen-Wen Cindy Chen, Veronica Ashton, Alicia K. Campbell, Dejan Milentijevic, W. Frank Peacock

**Affiliations:** 1 Northwell Health at Lenox Hill Hospital, New York, New York, United States; 2 The Institute of Health Systems Science at the Feinstein Institutes for Medical Research, Manhasset, New York, United States; 3 The Donald and Barbara Zucker School of Medicine at Hofstra/Northwell, Hempstead, New York, United States; 4 Janssen Scientific Affairs, LLC, Titusville, New Jersey, United States; 5 Henry JN Taub Department of Emergency Medicine, Baylor College of Medicine, Houston, Texas, United States

**Keywords:** anticoagulants, COVID-19, hospitalization, risk factors, thromboembolism

## Abstract

**Background** Most symptoms of coronavirus 2019 (COVID-19) are mild; however, some patients experience cardiovascular complications, including thromboembolic events and death. Data are needed to better inform prevention and treatment of these events. This analysis was designed to describe patient characteristics, medication use, thromboembolic events, and all-cause mortality in hospitalized COVID-19 patients in the United States.

**Methods** This retrospective, observational cohort study identified adults hospitalized with COVID-19 (January 21, 2020–January 07, 2021) in the deidentified Optum COVID-19 Electronic Health Records dataset. Thromboembolic events and all-cause mortality were collected at any time during the variable follow-up period (up to 50 weeks).

**Results** Of 181,995 COVID-19 patients who met eligibility criteria, 40,524 (22.3%) were hospitalized with COVID-19. Hospitalized patients had a mean age of 63 years and a Quan–Charlson comorbidity index of 1.3. Anticoagulants were used in 89.2% of patients during hospitalization and in 18.7% of postdischarge patients. Of hospitalized patients, 17.6% had a thromboembolic event during the entire follow-up period (mean time to the first event of 15 days), of whom 13.4% had an event during hospitalization; of discharged patients, 4.3% had a thromboembolic event (mean time from discharge to event of 43 days). Death during the follow-up period was reported in 15.0% of patients.

**Conclusions** In this large, observational cohort study, patients hospitalized with COVID-19 had high rates of thromboembolic events during hospitalization and in the postdischarge period; mortality was also high in this population. Anticoagulant use was common during hospitalization. These findings support further studies to optimize in-hospital and extended prophylaxis for hospitalized COVID-19 patients.

## Graphical abstract

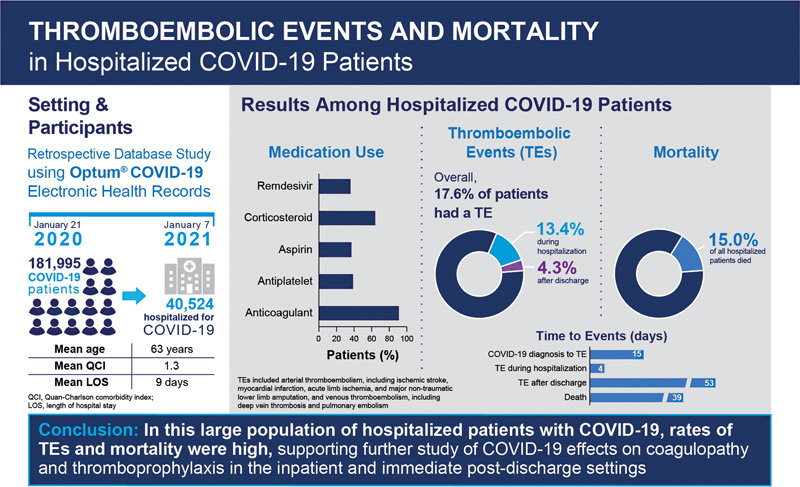

## Introduction

Coronavirus 2019 (COVID-19), an infectious disease caused by the severe acute respiratory syndrome coronavirus 2 (SARS-CoV-2), has rapidly spread throughout the world and overwhelmed health care resources. Most COVID-19 cases result in mild symptoms, such as fever, cough, fatigue, and shortness of breath; however, cardiovascular complications, including arterial thromboembolism (ATE) and venous thromboembolism (VTE), have been observed. 1 A recent meta-analysis reported the prevalence of COVID-19–related ATE and VTE to be 4.0% (95% confidence interval [CI]: 2.0–6.5%) and 14.7% (95% CI: 12.1–17.6%), respectively, with a higher VTE prevalence reported in critically ill patients (23.2% [95% CI: 17.5–29.6%]). 2 A network cohort study found lower but similar 90-day cumulative incidence rates for ATE and VTE among COVID-19 patients (0.1–0.8% and 0.2–0.8%), with increased rates among those hospitalized (3.1 and 4.6%). 3

Coagulopathy markers, including thrombocytopenia and elevated D-dimer, fibrin degradation products, prothrombin time, and partial thromboplastin time, have been associated with COVID-19 thromboembolic events and mortality. 4 5 SARS-CoV-2 infects endothelial cells through the angiotensin-converting enzyme 2 receptor, leading to thromboembolism. 4 6 7 Although the mechanistic understanding continues to evolve, preliminary reports suggest that a complex interplay of inflammatory cytokines (e.g., interleukins [IL], interferons, tumor necrosis factors, and endothelial cell dysfunction) triggers a coagulation cascade leading to hemostatic abnormalities, intravascular coagulopathy, formation of pulmonary microthrombi, and alteration of cardiac biomarkers and platelet function. 6 8 In particular, the correlation between IL-6 and fibrinogen levels supports the theory of inflammatory thrombosis. 9

To understand the risk of ATE and VTE in patients with COVID-19, a risk profile composed of characteristics, treatment journeys, and clinical outcomes among patients with COVID-19 is essential. Three of four components of metabolic syndrome (hypertension, diabetes, and hyperlipidemia) were associated with ATE in COVID-19 patients. 7 In addition, new-onset atrial fibrillation is common in patients with COVID-19, occurring in 7.4% of patients in a large meta-analysis and may contribute to ATE. 10 Older age (≥65 years); active smoking; history of stroke; low and high body mass index (BMI); and elevated D-dimer, platelet count, or C-reactive protein levels have been shown to be risk factors for VTE in patients with COVID-19. 5 11 12 13 14 Moreover, patients in the intensive care unit (ICU) exhibit a distinct phenotype that includes in situ pulmonary thrombosis more frequently than non-ICU patients. 15 Despite varying outcomes in ICU and non-ICU patients, 16 the American Society of Hematology, 17 18 American College of Chest Physicians, 19 National Institutes of Health, 20 and International Society on Thrombosis and Haemostasis 21 suggest using prophylactic anticoagulation in all hospitalized patients based on low-quality evidence. 22 23 Guidelines published by the Global COVID-19 Thrombosis Collaborative Group 6 and the VAS-European Independent Foundation in Angiology/Vascular Medicine 24 consider risk factors, particularly comorbidities, in determining the need for VTE prophylaxis. Early observational evidence suggests that prophylactic anticoagulation was rapidly implemented in hospitalized patients with COVID-19, a step that was associated with reductions in mortality. 25 Recently reported randomized trials support the use of therapeutic thromboprophylaxis over standard thromboprophylaxis in medical ward COVID-19 inpatients, but not in those with critical illness. 26 27 28

Although evidence on the management of COVID-19–related symptoms is increasing, additional knowledge is needed to inform the incidence of thromboembolic events in hospitalized patients with COVID-19. Therefore, the primary objectives of this study were to evaluate a large cohort of patients hospitalized with COVID-19 for demographic and clinical characteristics, use of relevant anticoagulant and other medications, and occurrence of thromboembolic events and all-cause mortality during and after hospitalization.

## Methods

### Data Source

This study was conducted using the longitudinal and low-latency Optum COVID-19 Electronic Health Records (EHR) dataset. Leveraging the deidentified Optum EHR database, COVID-19 patients were included in this Optum COVID-19 EHR dataset based on COVID-19 diagnosis codes and laboratory tests. All data elements in the Optum EHR database, including patient demographics, outpatient visits, coded diagnostic procedures, medications, laboratory results, hospitalizations, clinical notes, and patient outcomes from a network of health care provider organizations across the United States, were included in this COVID-19 dataset. As of January 7, 2021, there were 3.6 million patients in this dataset.

### Research Ethics

All study data were accessed with protocols compliant with U.S. patient confidentiality requirements, including the Health Insurance Portability and Accountability Act of 1996. Because this study consisted of secondary data analyses of deidentified patient records, the study did not constitute human subjects research and was exempt from Institutional Review Board registration and review requirements under the U.S. Federal Policy for the Protection of Human Subjects (also known as the “Common Rule”).

### Study Design and Study Population

**Fig. 1:**
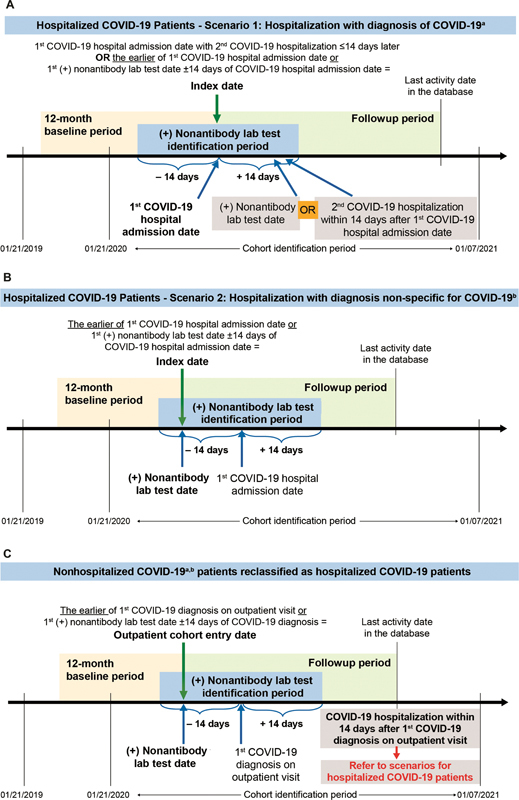
Study design for ( **A** ) patients hospitalized with a diagnosis of COVID-19, ^a^ ( **B** ) patients hospitalized with a diagnosis nonspecific for COVID-19, ^b^ and ( **C** ) nonhospitalized COVID-19 patients ^a,b^ who are hospitalized within 14 days for COVID-19. Examples of timelines are given, noting that patients whose index dates were later in the cohort identification period (e.g., November and December 2020) would have shorter available times for follow-up. COVID-19, coronavirus 2019; ICD-10-CM, *International Classicification of Diseases, 10th Revision, Clinical Modification* . ^a^ Defined as ICD-10-CM: U07.1, B97.29, B97.21, or B34.2. ^b^ Defined as ICD-10-CM: J12.81, J12.89, J20.8, J22, J40, J80, J98.8, A41.89, R05, R06.02, or R50.9.

This retrospective, observational cohort study analyzed the Optum EHR data of adult, hospitalized, COVID-19 patients for the 50-week period between January 21, 2020 and January 7, 2021 (cohort identification period; Fig. 1 ).

Adult patients (age ≥18 years) who had a hospital admission on or after January 21, 2020 and fit into one of the following two scenarios were included in the study: (1) having a coronavirus-related *International Classification of Diseases, 10th Revision, Clinical Modification* (ICD-10-CM) code (U07.1, B97.29, B97.21, B34.2) in any diagnosis position during a hospitalization; AND a positive COVID-19–specific nonantibody laboratory test ( Supplemental Table S1 ) within ± 14 days of the hospital admission date OR having a second hospitalization with a diagnosis of COVID-19 (ICD-10-CM: U07.1, B97.29, B97.21, or B34.2) in any diagnosis position within 14 days after the admission date of the first hospitalization ( Fig. 1A ); OR (2) having a code nonspecific for COVID-19 (ICD-10-CM: J12.81, J12.89, J20.8, J22, J40, J80, J98.8, A41.89, R05, R06.02, R50.9) in any diagnosis position AND a positive COVID-19–specific nonantibody laboratory test ( Fig. 1B and Supplemental Table S1 ) within ± 14 days of the hospital admission date. These inclusion criteria were used to ensure that the patients identified were truly hospitalized COVID-19 patients given the uncertainty in testing, coding, and diagnosis at the beginning of the pandemic. Nonhospitalized COVID-19 patients identified with a code for COVID-19 (ICD-10-CM: U07.1, B97.29, B97.21, B34.2, J12.81, J12.89, J20.8, J22, J40, J80, J98.8, A41.89, R05, R06.02, or R50.9) who were hospitalized for COVID-19 within 14 days following the first outpatient COVID-19 diagnosis were reclassified as hospitalized COVID-19 patients ( Fig. 1C ). Throughout this manuscript and unless the timing is specifically stated, outcomes described for “hospitalized patients” refer to outcomes occurring at any time during the follow-up period, both during the time that the patients were in-hospital and following their discharge. No other exclusion criteria were applied to the dataset.

The index date was defined as the date of the earliest evidence of confirmed COVID-19 on or after January 21, 2020 ( Fig. 1 ). Patients were also required to be aged 18 years or older as of the index date and to have had activity in the Optum EHR COVID database at least 12 months prior to the index date, which was defined as the baseline period. Patients with missing data on sex or birth year were excluded from the study. The period from the index date to the last activity date (up to January 7, 2021) in the database was considered as the follow-up period, which included both the hospitalization period and the time following discharge. Time of death was defined as the last day of the calendar month during which the patient died.

### Demographics, Medications of Interest, and Outcomes

- Patient demographic and clinical characteristics: Demographics were assessed on the index date and included age, sex, race, ethnicity, geographic region, insurance type, and month and year of index date. Clinical characteristics were assessed during the 12-month baseline period and included BMI and obesity class based on the last measure before the index date, comorbidities ( Supplemental Table S2 ), Quan–Charlson comorbidity index (QCI) score 29 ( Supplemental Table S3 ), and concomitant medication use ( Supplemental Table S4 ).
- Anticoagulant, anti-inflammatory/immunomodulatory, and thrombolytic medications: These medications of interest included anticoagulants, antiplatelets, tissue plasminogen activator, aspirin, antithrombin III, remdesivir, corticosteroids, and convalescent plasma ( Supplemental Table S5 ). The use of any of these treatments during the follow-up period was assessed in total and by the medication type. Treatment users were defined as patients with the use of any of the medications of interest during the follow-up period. Prior users were defined as patients with the use of any medications of interest during the follow-up period who also received any of the medications of interest within the 45 days prior to the index date. The number and proportion of treatment users and prior users of any medication in the class (overall and by each medication class) were assessed.
- Occurrence of thromboembolic events and all-cause mortality: Thromboembolic events included ATE, including ischemic stroke, myocardial infarction (MI), acute limb ischemia and major nontraumatic lower limb amputation, and VTE, including deep vein thrombosis (DVT) and pulmonary embolism (PE; Supplemental Table S6 ). The occurrence of thromboembolic events and time to the first event during the follow-up period were assessed in total and by each event of interest. All-cause mortality was determined as the number and proportion of patients who died during follow-up and the time to death was estimated from the date of death, defined in the Optum EHR COVID database as the last day of the calendar month during which the patient died.

*Exploratory Outcomes.* The following exploratory analyses were assessed:

- The length of hospital stay.
- Timing of thromboembolic event occurrence and medication use (during hospitalization or after discharge).
- The percentage of patients who received any of the medications of interest during hospitalization or after discharge.
- The percentage of patients who received any of the medications of interest within 45 days prior to the index date and who also received the same medication anytime during the follow-up period.
- The feasibility of incorporating COVID-19 risk stratification scores (i.e., the Northwell COVID-19 Survival [NOCOS] calculator 30 and the International Medical Prevention Registry on VTE and D-dimer [IMPROVE-DD] VTE Risk Score 31 32 ) was assessed by evaluating the availability of the risk score components in the database.

*Subgroup Analysis.* Among patients with a COVID-19 hospitalization, a subgroup analysis was performed to assess the occurrence of thromboembolic events in patients who were admitted to the ICU during the index hospitalization and those who were not admitted to the ICU.

### Data Analysis

Descriptive analyses were conducted for all study cohorts using univariate statistics including frequencies, percentages, means, standard deviations (SDs), medians, and interquartile range (IQR). No formal statistical tests or comparative assessments among patient groups or medications were performed in this study.

## Results

### Patient Demographic and Clinical Characteristics

**Fig. 2:**
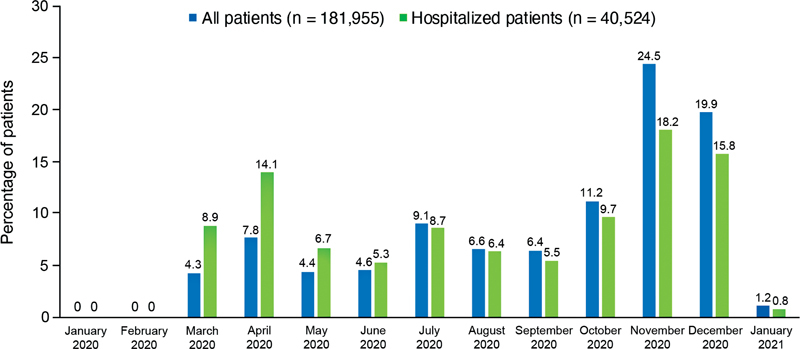
Month of index COVID-19 diagnosis for all patients and hospitalized patients. COVID-19, coronavirus 2019.

A total of 1,246,067 patients who had one or more EHR encounter with a code for COVID-19 in any diagnosis position during the cohort identification period were identified. After all eligibility criteria were applied, 181,995 patients were included in the overall cohort of COVID-19 patients, with a mean (SD) age of 50.0 (18.7) years and mean (SD) QCI score of 0.6 (1.4) ( Supplemental Table S7 ). Of these COVID-19 patients, 40,524 (22.3%) were hospitalized ( Table 1 ). During the period from January 2020 to January 2021, hospital admissions of these COVID-19 patients were highest during November, December, and April of 2020 ( Fig. 2 ). In the subset of patients with a COVID-related hospitalization, which is the focus of this manuscript, the mean (SD) age was 63.4 (17.4) years and nearly 20% of patients were African American ( Table 1 ). Among the 62% of patients with a BMI measure available, mean (SD) BMI was 32.3 (8.7) kg/m ^2^ . The mean (SD) QCI score was 1.3 (2.0) in hospitalized patients, with hypertension, hyperlipidemia, and diabetes being the most common comorbidities. Prior ischemic stroke and MI occurred in 3.0% of hospitalized patients, while prior DVT occurred in 1.9% and prior PE occurred in 1.3% ( Table 1 ). Baseline medication use was generally consistent with the presence of comorbidities ( Table 2 ). The highest reported medication used over the 12 months prior to the index date was antibiotics (34.6%), followed by antihypertensives (27.5%) and antihyperlipidemics (27.2%). Anticoagulants were used in 22.4% of hospitalized patients during the baseline period.

**Table 1:** Demographics and baseline a clinical characteristics of hospitalized COVID-19 patients

Number of patients	40,524
Age, years	
Mean (SD)	63.44 (17.36)
Age group, years, *n* (%)	
18–24	869 (2.14)
25–34	2,322 (5.73)
35–44	3,067 (7.57)
45–54	5,067 (12.50)
55–64	8,208 (20.25)
65+	20,991 (51.80)
Sex, *n* (%)	
Male	20,193 (49.83)
Female	20,331 (50.17)
Race, *n* (%)	
African American	7,912 (19.52)
Asian	864 (2.13)
Caucasian	25,580 (63.12)
Other/unknown	6,168 (15.22)
Ethnicity, *n* (%)	
Hispanic	5,167 (12.75)
Not Hispanic	31,918 (78.76)
Unknown	3,439 (8.49)
Geographic region, *n* (%)	
Northeast	10,174 (25.11)
West	3,100 (7.65)
Midwest	15,945 (39.35)
South	10,004 (24.69)
Other/unknown	1,301 (3.21)
Insurance type, *n* (%)	
Commercial	10,178 (25.12)
Medicaid	2,983 (7.36)
Medicare	9,326 (23.01)
Others	11,813 (29.15)
Uninsured	436 (1.08)
Unknown	1,355 (3.34)
Missing	4,433 (10.94)
BMI, kg/m ^2^	
Patients with BMI measure, *n* (%)	25,178 (62.13)
Mean BMI (SD) b	32.27 (8.70)
BMI category, *n* (%) b	
Underweight	379 (1.51)
Normal	4,143 (16.45)
Overweight	6,737 (26.76)
Obese	13,919 (55.28)
Obesity class, *n* (%) b	
Class 1 (BMI 30.0–34.9 kg/m ^2^ )	6,127 (24.33)
Class 2 (BMI 35.0–39.9 kg/m ^2^ )	3,849 (15.29)
Class 3 (BMI ≥40 kg/m ^2^ )	3,943 (15.66)
QCI, mean (SD)	1.28 (1.99)
Individual comorbidity, *n* (%)	
Hypertension	18,525 (45.71)
Hyperlipidemia	14,197 (35.03)
Diabetes	10,716 (26.44)
Anemia	6,342 (15.65)
Chronic kidney disease	6,133 (15.13)
Osteoarthritis	5,393 (13.31)
Nonalcoholic fatty liver disease	5,272 (13.01)
Depression	4,874 (12.03)
Thyroid disease	4,811 (11.87)
Anxiety	4,799 (11.84)
Congestive heart failure	4,719 (11.64)
Chronic obstructive pulmonary disease	4,339 (10.71)
Sleep apnea	3,597 (8.88)
Asthma	3,209 (7.92)
Cancer c	2,932 (7.24)
Prior stroke/transient ischemic attack	2,442 (6.03)
Peripheral vascular disease	1,785 (4.40)
Old MI	1,770 (4.37)
Osteoporosis	1,510 (3.73)
Rheumatoid arthritis	728 (1.80)
Liver cirrhosis	514 (1.27)
Stable angina	454 (1.12)
Unstable angina	212 (0.52)
Uveitis	39 (0.10)
Prior thromboembolic event, *n* (%)	
Ischemic stroke	1,226 (3.03)
MI	1,195 (2.95)
DVT	761 (1.88)
PE	518 (1.28)
ALI	124 (0.31)
Major nontraumatic lower limb amputation	160 (0.39)

Abbreviations: ALI, acute limb ischemia; BMI, body mass index; COVID-19, coronavirus 2019; DVT, deep vein thrombosis; MI, myocardial infarction; PE, pulmonary embolism; QCI, Quan–Charlson comorbidity index; SD, standard deviation.

a The baseline period was defined as the 12 months prior to the index date.

b Mean BMI, BMI categories, and obesity classes were identified among hospitalized patients with a BMI measure available ( *n* = 25,178).

c A diagnosis for cancer required two diagnosis codes for the same type of cancer at least 30 days apart.

**Table 2:** Baseline medication use in hospitalized COVID-19 patients

Baseline medication use, a *n* (%)	Hospitalized patients ( *n* = 40,524)
Antibiotics	14,007 (34.56)
Antihypertensives	11,159 (27.54)
Antihyperlipidemics	11,004 (27.15)
Beta-blockers	9,486 (23.41)
Anticoagulants	9,095 (22.44)
Corticosteroids	8,927 (22.03)
Antidiabetics	8,731 (21.55)
Diuretics	7,978 (19.69)
Anti-inflammatory agents	7,209 (17.79)
Antiplatelet agents	6,974 (17.21)
Calcium channel blockers	6,844 (16.89)
Antiarrhythmics	6,821 (16.83)
Antidepressants	6,813 (16.81)
Antianxiety agents	5,057 (12.48)
Antineoplastic agents	1,345 (3.32)

Abbreviation: COVID-19, coronavirus 2019.

a The baseline period was defined as the 12 months prior to the index date.

### Results During Follow-Up

For all hospitalized patients, the overall mean (SD) time from index to end of follow-up was 80.3 (82.8) days, with a median (IQR) of 43 (18–123) days.

### Relevant Medication Use

Nearly all (94.8%) hospitalized patients used a medication of interest during follow-up ( Table 3 ). Anticoagulants were used in 90.9% of hospitalized patients, with low molecular weight heparins (LMWHs) being the most frequently used anticoagulant (70.2%). Among hospitalized patients, 38.6% received antiplatelet agents, 63.7% received corticosteroids, and 35.5% received remdesivir. Convalescent plasma was used in 8.4% of hospitalized patients. Nearly, 20% of patients used an anticoagulant after discharge ( Table 4 ).

**Table 3:** Use of medications of interest during follow-up in hospitalized COVID-19 patients

	Medication use during follow-up	Both prior use a and follow-up use b
Number of patients	40,524	40,524
Medication use, *n* (%)		
Any of the medications of interest c	38,403 (94.77)	5,014 (12.37)
Anticoagulants		
Any anticoagulants d	36,824 (90.87)	2,773 (6.84)
Vitamin K antagonists	1,291 (3.19)	229 (0.57)
Factor Xa inhibitors	7,744 (19.11)	651 (1.61)
UFH	13,634 (33.64)	983 (2.43)
LMWH	28,458 (70.23)	778 (1.92)
DTIs	344 (0.85)	24 (0.06)
Antiplatelets e	15,635 (38.58)	1,585 (3.91)
Aspirin	14,798 (36.52)	578 (1.43)
tPA	2,448 (6.04)	85 (0.21)
Remdesivir	14,403 (35.54)	26 (0.06)
Corticosteroids	25,809 (63.69)	2,083 (5.14)
Convalescent plasma	3,384 (8.35)	0
Antithrombin III	4 (0.01)	0

Abbreviations: COVID-19, coronavirus 2019; DTI, direct thrombin inhibitor; LMWH, low molecular weight heparin; tPA, tissue plasminogen activator; UFH, unfractionated heparin.

a Prior use was defined as documentation of the medication(s) of interest during the 45-day period prior to the index date.

b Follow-up use was defined as documentation of the medication(s) of interest on or after the index date.

c The medications of interest included anticoagulants, antiplatelets, aspirin, tPA, remdesivir, corticosteroids, convalescent plasma, and antithrombin III.

d Anticoagulants included vitamin K antagonists, factor Xa inhibitors, UFH, LMWH, and DTIs.

e Antiplatelet medications did not include aspirin.

**Table 4:** Thromboembolic events and mortality during follow-up in hospitalized COVID-19 patients

Number of patients	40,524
Occurrence of thromboembolic events, *n* (%)	
Any of the thromboembolic events of interest a	7,113 (17.55)
Ischemic stroke	1,544 (3.81)
MI	3,119 (7.70)
DVT	1,761 (4.35)
PE	1,849 (4.56)
ALI	89 (0.22)
Major nontraumatic lower limb amputation	118 (0.29)
Time to first event (days), b mean (SD)	
Any of the thromboembolic events of interest a	15.0 (34.2)
Ischemic stroke	23.7 (47.1)
MI	12.3 (31.3)
DVT	22.3 (41.2)
PE	14.6 (30.1)
ALI	38.8 (57.9)
Major nontraumatic lower limb amputation	33.6 (56.0)
Death c	
Patients who died during follow-up, *n* (%)	6,077 (15.0)
Time to death (days), mean (SD)	39.0 (32.2)

Abbreviations: ALI, acute limb ischemia; COVID-19, coronavirus 2019; DVT, deep vein thrombosis; MI, myocardial infarction; PE, pulmonary embolism; SD, standard deviation.

a The thromboembolic events of interest included ischemic stroke, MI, DVT, PE, ALI, and major nontraumatic lower limb amputation.

b Time to first event was examined among patients with the event.

c In the Optum COVID-19 dataset, the death date was defined as the last day of the calendar month during which the patient died. Therefore, the results reported here are an estimate.

### Thromboembolic Events and Mortality

**Fig. 3:**
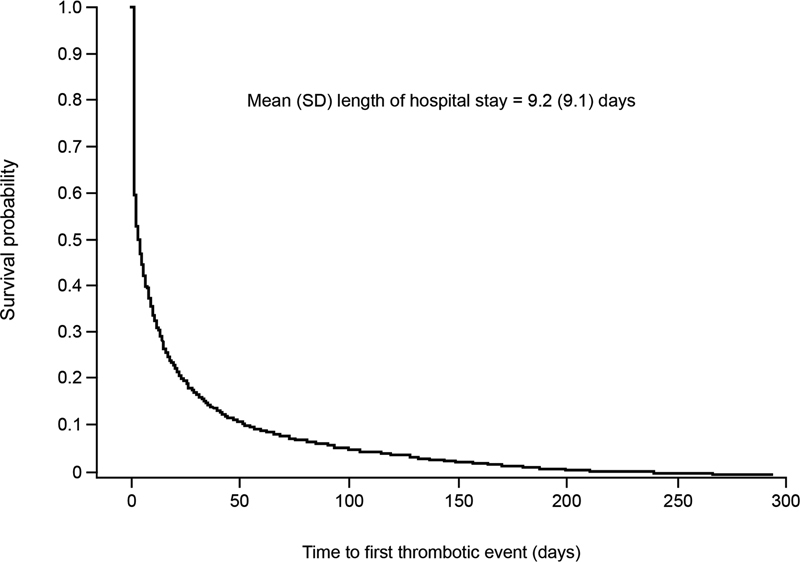
Time to first thromboembolic event. The median (interquartile range) for thromboembolic events to accrue was 4 (1, 17) days, and the probability of having the first thromboembolic event at day 9 (mean duration of hospital stay) was 35.9%. SD, standard deviation.

Among patients with a COVID-19 hospitalization, thromboembolic events of interest occurred in 7,113 patients (17.6%; Table 5 ), with 13.4% experiencing an event during hospitalization and 4.3% experiencing an event only after hospital discharge ( Table 4 ). Time to first thromboembolic event is shown in Fig. 3 . During hospitalization, MI was the most common thromboembolic event, followed by PE, DVT, and ischemic stroke. After discharge, the proportions of patients with MI, DVT, PE, and ischemic stroke were similar.

**Table 5:** Exploratory analysis: thromboembolic events and medication use during hospitalization and after discharge (not mutually exclusive)

	During hospitalization	After discharge (includes patients who had a thromboembolic event only after discharge from index hospitalization)
Total number of patients a	40,524	37,541
Occurrence of thromboembolic events, *n* (%)		
Any of the thromboembolic events of interest b	5,430 (13.40)	1,603 (4.27)
Ischemic stroke	1,080 (2.67)	443 (1.18)
MI	2,497 (6.16)	574 (1.53)
DVT	1,205 (2.97)	542 (1.44)
PE	1,372 (3.39)	465 (1.24)
ALI	47 (0.12)	39 (0.10)
Major nontraumatic lower limb amputation	86 (0.21)	32 (0.09)
Time to first event (days), mean (SD) c		
Any of the thromboembolic events of interest b	3.9 (5.7)	53.4 (56.4)
Ischemic stroke	4.4 (7.3)	71.6 (66.3)
MI	3.2 (5.2)	52.6 (56.6)
DVT	5.8 (8.5)	59.7 (57.8)
PE	4.6 (6.3)	44.5 (47.8)
ALI	3.6 (6.0)	83.5 (63.8)
Major nontraumatic lower limb amputation	6.6 (7.1)	105.9 (65.4)
Medication use, *n* (%)		
Any of the medications of interest d	37,917 (93.57)	9,582 (25.52)
Anticoagulants		
Any anticoagulants e	36,146 (89.20)	7,035 (18.74)
Vitamin K antagonist	1,143 (2.82)	475 (1.27)
Factor Xa inhibitor	6,939 (17.12)	2,425 (6.46)
UFH	12,213 (30.14)	2,853 (7.60)
LMWH	27,385 (67.58)	3,388 (9.02)
DTIs	286 (0.71)	77 (0.21)
Antiplatelets f	14,490 (35.76)	3,612 (9.62)
Aspirin	13,666 (33.72)	3,269 (8.71)
tPA	2,065 (5.10)	483 (1.29)
Remdesivir	13,772 (33.98)	763 (2.03)
Corticosteroids	23,992 (59.20)	4,659 (12.41)
Convalescent plasma	3,302 (8.15)	29 (0.08)
Antithrombin III	2 (0.00)	2 (0.01)

Abbreviations: ALI, acute limb ischemia; DTI, direct thrombin inhibitor; DVT, deep vein thrombosis; LMWH, low molecular weight heparin; MI, myocardial infarction; PE, pulmonary embolism; SD, standard deviation; UFH, unfractionated heparin; tPA, tissue plasminogen activator.

a Patients who died or were censored during hospitalization were excluded from the total number of patients after discharge.

b The thromboembolic events of interest included ischemic stroke, MI, DVT, PE, ALI, and major nontraumatic lower limb amputation.

c Time to first event was examined among patients with the event.

d The medications of interest included anticoagulants, antiplatelets, aspirin, tPA, remdesivir, corticosteroids, convalescent plasma, and antithrombin III.

e Anticoagulants included vitamin K antagonists, factor Xa inhibitors, UFH, LMWH, and DTIs.

f Antiplatelet medications did not include aspirin.

The mean (SD) time from COVID-19 diagnosis to thromboembolic events for all hospitalized patients was 15.0 (34.2) days, and the mean (SD) length of hospital stay was 9.2 (9.1) days. For patients with an event during hospitalization, the mean (SD) time to thromboembolic event was 3.9 (5.7) days, with the shortest time to MI (3.2 [5.2] days) and longest times to DVT (5.8 [8.5] days) and major nontraumatic lower limb amputation (6.6 [7.1] days). Of patients who were discharged, the mean (SD) follow-up period was 70.7 (82.0) days after discharge. For patients who had a thromboembolic event only after discharge from the hospital, the mean (SD) time to first event was 53.4 (56.4) days after COVID-19 diagnosis and ranged from 44.5 (47.8) days for PE to 105.9 (65.4) days for major nontraumatic lower limb amputation. The mean (SD) time from discharge to event was 43 (56) days.

Death occurred in 6,077 (15.0%) of hospitalized COVID-19 patients ( Table 5 ). The estimated mean (SD) time to death was 39.0 (32.2) days for all hospitalized patients.

### Risk Stratification Scores

In the exploratory analysis to determine the feasibility of assessing risk stratification scores, a high proportion of hospitalized patients (>95%) had values available for each of the elements of the NOCOS calculator, except for serum sodium levels, which were available in 65.8% of the patients ( Supplemental Table S8 ). After excluding patients with data not meeting the requirement for the NOCOS calculator, 30 the complete data of 22,557 (55.7%) hospitalized COVID-19 patients were entered into the NOCOS calculator to assess their survival probability. These patients had a mean 62.8% probability of survival. The IMPROVE-DD VTE risk score was calculated for all hospitalized patients. If a component of IMPROVE-DD VTE risk score was not found during index hospitalization, a value of 0 was assigned. Among all hospitalized patients, the mean (SD) IMPROVE-DD VTE risk score was 3.0 (2.0). About three-quarters of the hospitalized patients had IMPROVE-DD VTE risk score ≥2 ( *n* = 30,060).

### Subgroup Analysis

An ICU stay was recorded for 21.3% of hospitalized patients, of which 24.1% ( *n* = 2,083) had a thromboembolic event of interest ( Supplemental Table S9 ). The time to first thromboembolic event ranged from 9 days for MI to more than 1 month for major nontraumatic lower limb amputation in hospitalized patients admitted to the ICU.

## Discussion

Using a large EHR database of patients in the United States, we identified a cohort of COVID-19 patients and assessed patient characteristics, relevant medication use, and the occurrence of thromboembolic events and all-cause mortality. There are three important findings from our work. First, nearly one-quarter of all identified COVID-19 patients were hospitalized, providing a cohort of more than 40,000 patients with a COVID-19 hospitalization, which is substantially larger than previously reported real-world analyses. 33 34 These patients had risk factors for more severe COVID-19, including older age, African-American race, and a high QCI, which is indicative of comorbidity burden. 11 13 Although prior thromboembolic events were infrequently reported, 22.4% of hospitalized patients had received anticoagulants during the 12-month baseline period prior to their COVID-19 diagnosis. Second, thromboembolic events and all-cause mortality occurred in 17.6 and 15.0% of hospitalized patients, respectively. Thromboembolic events occurred in 13.4% of patients during hospitalization and were numerically higher among patients in the ICU (24.1%) than those who did not have an ICU stay (15.8%). The rate of thromboembolism was consistent with the rates of ATE and VTE reported in recent studies. 2 16 Third, the rate of postdischarge thromboembolic events of 4.3% was higher than previously reported among smaller cohorts 35 36 37 38 but similar to the larger CORE-19 registry. 34

This observational and descriptive study utilized a geographically diverse database spanning all 50 states to provide results that are generalizable to all COVID-19 patients across the United States. In addition, this database has a lag time of only 1 to 2 months, allowing near real-time analysis of real-world data. COVID-19 patients were identified using nonantibody tests, which have very high specificity and limited false-positive results.

A cohort study of 1,351 hospitalized COVID-19 patients in Michigan provides consistent results to ours for mortality while examining the use of prophylactic versus treatment-dose anticoagulants. 33 Overall, anticoagulation was administered to 88% of patients, with more intensive therapy to older patients, those with longer hospital stays, more comorbidities, and more severe disease. Prophylactic VTE treatment was reported for 83.4% of patients, while 16.2% received treatment-dose anticoagulation. The rate of confirmed VTE was 1.3% after day 2 of hospitalization. The in-hospital mortality rate was 15.8% for patients who received prophylactic anticoagulants and 33.8% for those who received treatment-dose anticoagulants, but both methods of anticoagulation were associated with lower in-hospital mortality compared with no anticoagulation. 33 In another study using a multihospital integrated health care network in New York, a retrospective cohort study identified 9,407 hospitalized COVID-19 patients. 5 Most patients were aged older than 60 years (63.8%), male (59.3%), and had hypertension (59.9%), along with other comorbidities. During hospitalization, 18.6% of patients received treatment-dose thromboprophylaxis and 71.0% received prophylactic dose, while 10.4% received no initial hospital thromboprophylaxis. VTE occurred in 2.9% of patients, with a higher rate in the ICU versus medical ward (4.9 vs. 2.4%). Of those who developed at least 1 VTE, 10.2% did not receive anticoagulation prior to diagnosis, 65.7% received prophylactic dose, and 24.1% received treatment dose. The overall VTE or mortality rate was 26.1% and followed similar trends as the VTE rate for patient sex, hospital location, and D-dimer levels. VTE or mortality increased with increasing comorbidity burden. These results provide support for in-hospital thromboprophylaxis. 5 Given the high rate of thromboembolic events, current guidelines from health organizations and hematologic societies suggest the use of prophylactic anticoagulants, with LMWH as drug of choice, for hospitalized COVID-19 patients. 6 17 18 19 20 21 24 Our analysis indicates that these guidelines are being followed, with 95% of hospitalized patients receiving anticoagulants and LMWH used in 70% of patients. Recent randomized trials suggest advantages of therapeutic-dose thromboprophylaxis over standard thromboprophylaxis in medical ward COVID-19 inpatients, especially those with elevated D-dimer levels. 25 26 28 This advantage was not seen in critically ill COVID-19 in-patients. 26 27

The effect of COVID-19 on postdischarge thromboembolism, and associated continued use of thromboprophylaxis, is less clear. 39 Our study found a posthospital discharge rate of thromboembolism of 4.3%. This rate is similar to the largest prospective study of postdischarge rates of thromboembolism and mortality in over 4,900 hospitalized COVID-19 patients in the CORE-19 registry. 34 During hospitalization, thromboprophylaxis was reported for 82.3% of patients, while postdischarge thromboprophylaxis was reported for 12.7% of patients, with a rate of 21.5% postdischarge thromboprophylaxis for patients with an IMPROVE-DD VTE risk score ≥4. Postdischarge VTE and ATE occurred in 1.6 and 1.7% of patients, respectively, and postdischarge all-cause mortality was 4.8%. Key predictors of postdischarge thromboembolic events in the CORE-19 registry were advanced age (>75 years), cardiovascular risk factors, chronic kidney disease, IMPROVE-DD VTE score ≥4, and an ICU stay. The risk of major thromboembolic events and death was reduced by 46% in patients who received postdischarge anticoagulants. 34 The Northwell Health System policy advocated for the extended use of thromboprophylaxis for 30 days after hospitalization based on randomized trials in high-risk medically ill patients. 40 41 In our study, 4.3% of patients developed thromboembolic events only after discharge despite anticoagulant use in 18.7% of patients, although it is not known if the events occurred in patients receiving anticoagulants. The recently published MICHELLE trial using both the IMPROVE-DD VTE tool with a score of 4 or more or a score of 2 to 3 and elevated D-dimer to select high-risk COVID-19 inpatients in the postdischarge period revealed a 6% absolute risk reduction (67% relative risk reduction; relative risk 0.33; 95% CI: 0.12–0.90; *p* = 0.0293) of major thromboembolic events and cardiovascular death favoring postdischarge extended thromboprophylaxis with rivaroxaban over no anticoagulation. 42

Risk stratification tools are helpful in managing the influx of pandemic patients by identifying more severely ill patients and advising therapeutic decisions. We assessed the feasibility of determining the NOCOS calculator and IMPROVE-DD VTE risk score based on the presence of score characteristics in our database population. Both risk assessment models have been validated in hospitalized COVID-19 patients. 30 31 32 Most patients (>95%) had some data available for the components of the NOCOS calculator; however, only 55.7% had complete NOCOS data. Using these data, their mean survival probability was 62.8%, which indicates a seriously ill population but also overestimates the observed mortality rate of 15%, suggesting that the NOCOS calculator was not feasible for use in this population. Fewer patients (0.3–74.4%) had data available for components of the IMPROVE-DD VTE risk score. Using a value of 0 for missing data, which may underestimate VTE risk, results of the IMPROVE-DD VTE score for all hospitalized COVID-19 patients suggested an overall moderate-to-high risk of VTE.

Inherent to claims analyses, data coding is not without its limitations. Given the observational and descriptive study design, causal inferences between treatment and outcomes were not assessed. Some limitations of the data sources include the fact that health care provider organizations must participate in the EHR network to be included so that data on the geographic distribution of the cohort may be impacted by geographic differences in network participation. Second, patients may receive laboratory tests based on their risk for certain conditions, and thus, these patients may have different demographic and clinical characteristics than patients who do not receive such tests. As atrial fibrillation is known to occur in COVID-19 patients, the lack of data on this condition may have impacted the outcomes. Third, death data were not available for all patients. The dataset used in this study also included a very small proportion of uninsured patients. We also note that the study was conducted using data from 2020 and represents COVID-19 at that time. The effect of differences in viral variants, COVID-19 patient populations, and treatments over time on thromboembolic events was also not determined. 43 44 In addition, we could not determine if death occurred during hospitalization or after discharge due to limitations around death date in the dataset. Because the death date was defined as the last day of the calendar month during which the patient died, time of death in this study is an estimation. Further, this analysis examined the number of patients with various thromboembolic events but did not count multiple occurrences of the same event for each patient. It is possible that events competed with each other and death in this cohort (as subclinical thrombotic disease) was found in 60% of COVID-19 patient autopsies. 45 Written prescriptions captured in EHR data do not necessarily indicate that the medication was taken. The use of aspirin, an over-the-counter medication in the United States, may also have been underestimated. Differences in patient characteristics and treatment objectives associated with each of the treatments of interest may limit interpretability of the results of this study.

Although this study is based on a population of patients that was earlier in the pandemic and may thus have been qualitatively different than patient populations at this time, the fundamental risk conditions for risk stratification and poor outcomes of hospitalized patients are strongly supported by this study. Particularly given the overall mortality rate of this population, results from our study demonstrate the value of leveraging large real-world datasets to characterize the thromboembolic complications of COVID-19, both during hospitalization and particularly in the posthospitalization period. Further high-quality studies exploring the relationship of baseline thromboprophylaxis with the incidence of COVID-19–related in-hospital VTE and posthospitalization VTE and death will be needed to establish the evidence base for informing the use of extended thromboprophylaxis following COVID-19 hospitalization.

## Conclusions

In a cohort of more than 40,000 hospitalized COVID-19 patients, thromboembolic events occurred in 18% and the mortality rate was 15%. Importantly, 4% of thromboembolic events developed only after discharge from the hospital. These findings support further study of the effects of COVID-19 on coagulopathy and optimization of thromboprophylaxis for patients hospitalized for COVID-19, both during hospitalization and in the immediate postdischarge period.

## Notes

**What is known on this Topic?**

- Thromboembolism is a substantial concern for hospitalized patients with COVID-19, and the effect appears to extend into the postdischarge period.
- Most treatment guidelines recommend prophylactic anticoagulation for hospitalized COVID-19 patients to lower the risk of thromboembolism and mortality.
- This study evaluated patient characteristics, medication use, thromboembolic events, and all-cause mortality in a large, observational cohort of more than 40,000 patients hospitalized with COVID-19 in the United States.

**What does this Paper Add?**

- In this study, 13.4% of patients experienced a thromboembolic event during hospitalization and 4.3% experienced a thromboembolic event only after discharge; 15% of patients died during follow-up.
- Among hospitalized COVID-19 patients, those who experienced a thromboembolic event were characterized by trends toward older age and increased comorbidity burden.
- The size and geographic distribution of this analysis are sufficiently robust as to describe what may be considered as the current standard of care in the management of patients suffering from COVID-19 and may be particularly relevant when institutions and professional societies consider the construction of guidelines and care pathways for this patient population.
