# Supplementary material for: Occurrence of Thromboembolic Events and Mortality Among Hospitalized Coronavirus 2019 Patients: Large Observational Cohort Study of Electronic Health Records
Source: TH Open. 2022 Nov 18;6(4):e408–20. doi: 10.1055/a-1937-9692 (PMC9674436; doi:10.1055/a-1937-9692)
Supplement: Supplementary file 1 — Supplementary Material [file 10-1055-a-1937-9692-s22080035.pdf]

Supplemental Materials

Supplemental Table S1 Codes for identification of COVID-19 patients[46–49]

Diagnosis codes (ICD-10-CM)		
Code	Description	CDC guidelines (April 2020 unless otherwise noted)
Confirmed COVID-19 diagnosis[a]		
U07.1	COVID-19	For a confirmed diagnosis, assign U07.1. If the provider documents “suspected,” “possible,” “probable,” or “inconclusive” COVID-19, do not assign code U07.1. For asymptomatic individuals who test positive for COVID-19, assign code U07.1.
Nonspecific coronavirus diagnoses		
B34.2	Coronavirus infection, unspecified	N/A
B97.21	SARS-associated coronavirus as the cause of diseases classified elsewhere	N/A
B97.29	Coronavirus as cause of disease classified elsewhere	CDC February 2020 guideline (prior to creation of U07.1): Assign B97.29 along with codes to describe the illness (e.g., J12.89, J20.8, J22, J40, J80, or J98.8)
Acute illnesses potentially due to COVID-19		
J12.81	Pneumonia due to SARS-associated coronavirus	N/A
J12.89	Other viral pneumonia	For a pneumonia case confirmed as due to COVID-19, assign codes U07.1, COVID-19, and J12.89, Other viral pneumonia
J20.8	Acute bronchitis due to other specified organisms	For a patient with acute bronchitis confirmed as due to COVID-19, assign codes U07.1 and J20.8, Acute bronchitis due to other specified organisms
J22	Unspecified acute lower respiratory infection	If COVID-19 is documented as being associated with a lower respiratory infection, NOS, or an acute respiratory infection, NOS, codes U07.1 and J22, Unspecified acute lower respiratory infection should be assigned
J40	Bronchitis not specified as acute or chronic	Bronchitis NOS due to COVID-19 should be coded using code U07.1 and J40, Bronchitis, not specified as acute or chronic

 © 2022. The Author(s).

Supplemental Table S1 (*Continued*)

Diagnosis codes (ICD-10-CM)		
Code	Description	CDC guidelines<br>(April 2020 unless otherwise noted)
J80	Acute respiratory distress syndrome	For ARDS due to COVID-19, assign codes U07.1, and J80, ARDS
J98.8	Other specified respiratory disorders	If COVID-19 is documented as being associated with a respiratory infection, NOS, codes U07.1 and J98.8, Other specified respiratory disorders should be assigned
A41.89	Other specified sepsis	For COVID-19 infection that progresses to sepsis
Symptoms potentially due to COVID-19		
R05	Cough	For patients presenting with any signs/symptoms associated with COVID-19 but a definitive diagnosis has not been established
R06.02	Shortness of breath	
R50.9	Fever, unspecified	
Laboratory test codes (LOINC)		
Nonantibody tests	Description	
Antigen: 96119–3, 95942–9, 95209–3, 94558–4<br>PCR/nucleic acid detection: 94565–9, 95608–6, 95941–1, 95380–2, 95423–0, 95422–2, 96094–8, 95521–1, 94510–5, 95522–9, 94760–6, 95409–9, 94533–7, 95425–5, 94766–3, 94316–7, 94644–2, 94511–3, 94559–2, 95824–9, 94639–2, 94646–7, 94645–9, 96120–1, 94534–5, 96091–4, 94314–2, 96123–5, 94745–7, 94746–5, 94819–0, 94759–8, 95406–5, 94500–6, 94845–5, 94660–8, 94309–2, 94531–1, 95826–4, 94306–8, 94642–6, 94643–4, 94640–0, 94767–1, 94641–8, 94313–4, 94310–0, 94509–7, 96121–9, 94758–0, 95823–1, 94765–5, 94315–9, 96122–7, 94502–2, 94647–5, 94532–9, 94311–8, 94756–4, 94307–6, 94312–6, 94757–2, 94308–4, 95424–8, 94822–4, 95609–4, 95970–0, 94764–8	Name contains "COVID 19, COVID-19, SARS coronavirus 2, SARS CoV 2, SARS-CoV-2, 2019 novel coronavirus"	

Abbreviations: ARDS, acute respiratory distress syndrome; CDC, Centers for Disease Control and Prevention; COVID-19, coronavirus 2019; ICD-10-CM, *International Classification of Diseases*, *10th Revision*, *Clinical Modification*; N/A, not applicable; NOS, not otherwise specified; SARS-CoV-2, severe acute respiratory syndrome coronavirus 2.

[a]COVID-19–specific diagnosis codes (U) were introduced by the World Health Organization in late March 2020. Prior to that, the CDC recommended use of nonspecific coronavirus (B) and acutely ill diagnosis codes (J&R) to document COVID-19 infections. Z codes for screening or potential exposure to viral diseases (e.g., Z03.818, Z11.59, Z20.828) were not included as part of our COVID-19 definition because they are to be used as "rule out" codes, per CDC guidance.

 © 2022. The Author(s).

Supplemental Table S2 Codes for comorbid conditions

Disease of interest	ICD-10-CM
Anemia	D50.%–D64.%
Anxiety	F41.%
Asthma	J45.%
Acute MI	I21.%, I22.%
Old MI	I25.2%
Stable angina	I20.1%–I20.9%,
Unstable angina	I20.0%, I24.0%
Peripheral vascular disease	I70.%, I71.%, I73.1%, I73.8%, I73.9%, I77.1%, I79.0%, I79.2%, K55.1%, K55.8%, K55.9%, Z95.8%, Z95.9%
Congestive heart failure	I50.%
Cancer[a]	C00.%–C26.%, C30.%–C34.%, C37.%–C41.%, C43.%, C4A.%, C44.%–C58.%, C60.%–C76.%, C81.%–C85.%, C88.%, C90.%–C97.%
COPD	J41.%, J42.%, J43.%, J44.%
Depression	F32.%, F33.%, F34.1%
Diabetes	E10.%–E13.%
Hypertension	I10.0%–I16.0%
Hyperlipidemia	E78.0%, E78.1%, E78.2%, E78.4%, E78.5%
Liver cirrhosis	K70.3%, K74.%
Chronic kidney disease	N18.%
Nonalcoholic fatty liver disease	I12.%, I13.%, K76.0%
Osteoarthritis	M15.%–M19.%
Osteoporosis	M80.%, M81.%
Rheumatoid arthritis	M05.%, M06.%
Prior stroke/TIA	I61.%–I66.%, G45.%
Thyroid disease	E00.%–E07.%
Uveitis	H20.%

Abbreviations: COPD, chronic obstructive pulmonary disease; ICD-10-CM, *International Classification of Diseases, 10th Revision, Clinical Modification*; MI, myocardial infarction; TIA, transient ischemic attack.
[a]A diagnosis for cancer required 2 diagnosis codes for the same type of cancer at least 30 days apart.

 © 2022. The Author(s).

Supplemental Table S3 Codes for QCI

Comorbidities	ICD-10-CM codes[50]	Quan 2010 weight[29]
Myocardial infarction	I21.%, I22.%, I25.2%	0
Congestive heart failure	I09.9%, I11.0%, I13.0%, I13.2%, I25.5%, I42.0%, I42.5%–I42.9%, I43.%, I50.%, P29.0%	2
Peripheral vascular disease	I70.%, I71.%, I73.1%, I73.8%, I73.9%, I77.1%, I79.0%, I79.2%, K55.1%, K55.8%, K55.9%, Z95.8%, Z95.9%	0
Cerebrovascular disease	G45.%, G46.%, H34.0%, I60.%–I69.%	0
Dementia	F00.%–F03.%, F05.1, G30.%, G31.1%	2
Chronic pulmonary disease	I27.8%, I27.9%, J40.%–J47.%, J60.%–J67.%, J68.4%, J70.1%, J70.3%	1
Connective tissue/rheumatic disease	M05.%, M06.%, M31.5%, M32.%–M34.%, M35.1%, M35.3%, M36.0%	1
Peptic ulcer disease	K25.%–K28.%	0
Mild liver disease	B18.%, K70.0%–K70.3%, K70.9%, K71.3%–K71.5%, K71.7%, K73.%, K74.%, K76.0%, K76.2%–K76.4%, K76.8%, K76.9%, Z94.4%	2
Diabetes without chronic complications/mild to moderate	E10.0%, E10.1%, E10.6%, E10.8%, E10.9%, E11.0%, E11.1%, E11.6%, E11.8%, E11.9%, E12.0%, E12.1%, E12.6%, E12.8%, E12.9%, E13.0%, E13.1%, E13.6%, E13.8%, E13.9%, E14.0%, E14.1%, E14.6%, E14.8%, E14.9%	0
Paraplegia and hemiplegia	G04.1%, G11.4%, G80.1%, G80.2%, G81.%, G82.%, G83.0%–G83.4%, G83.9%	2
Renal disease	I12.0%, I13.1%, N03.2%–N03.7%, N05.2%–N05.7%, N18.%, N19.%, N25.0%, Z49.0%–Z49.2%, Z94.0%, Z99.2%	1
Diabetes with chronic complications	E10.2%–E10.5%, E10.7%, E11.2%–E11.5%, E11.7%, E12.2%–E12.5%, E12.7%, E13.2%–E13.5%, E13.7%, E14.2%–E14.5%, E14.7%	1
Any malignancy, including lymphoma and leukemia, except malignant neoplasm of skin	C00.%–C26.%, C30.%–C34.%, C37.%–C41.%, C43.%, C45.%–C58.%, C60.%–C76.%, C81.%–C85.%, C88.%, C90.%–C97.%	2
Moderate or severe liver disease	I85.0%, I85.9%, I86.4%, I98.2%, K70.4%, K71.1%, K72.1%, K72.9%, K76.5%, K76.6%, K76.7%	4
Metastatic solid tumor	C77.%–C80.%	6
AIDS/HIV	B20.%–B22.%, B24.%	4

Abbreviations: AIDS, acquired immunodeficiency syndrome; HIV, human immunodeficiency virus; ICD-10-CM, International *Classification of Diseases 10th Revision, Clinical Modification*; QCI, Quan–Charlson comorbidity index.
The table above outlines the updated QCI index. To quantify comorbidity, the QCI score is computed by adding the weights that are assigned to the specific diagnoses. Each diagnosis is only counted once. The minimum possible score is 0, and the maximum possible score is 24.

 © 2022. The Author(s).

Supplemental Table S4 Codes for concomitant medications

Medication class	GPI code starts with:	HCPCS codes
Antianxiety agents	57%	J1990, J3360, J2060, J1790, J1810, J3410
Antibiotics	01–08%, 1,600–1,629%, 1,699%	J0290, J0295, J2543, S0040, J0690, J1890, S0074, J0694, J0697, S0021, J0698, J0713, J0715, J0696, J0692, J0712, S0077, J2010, J1364, J0456, J2460, J2265, J0120, J0200, J0744, J1956, J2280, S0034, J1590, J0278, J1580, J1840, J1850, J3000, J3260, S0073, J0770, J1267, C9241, J1335, J2185, J3243, J0743, S0039
Antidepressants	58%	J1320, S0106
Antidiabetics	27%	J1815, J1817, E0784, S5550-S5571, G9147, S9353
Antihyperlipidemics	39%	N/A
Antihypertensives	36%	J0210, J2760, J0360, S0139, J1730, J2670
Antiplatelets	8,515%	J1245, J0130, J1327, J3246, C9460
Antineoplastic agents	21%	J8501-J9999
Antiinflammatory agents	66%	J1130, J1741, J1885, J2910, J1600, J9250, J9260, J8610, J0135, J1602, J1438, J0129, J2793, J0638, J3262
Beta-blockers	33%, 369920%, 369927%, 369988%	J1800, C9482
Calcium channel blockers	34%, 369915%, 369930%, 369967%, 369968%	C9248
Corticosteroids	22%, 8915%	J0702, J7624, J7622, J7633, J7627, J7634, J7626, J8540, J1094, J1100, J7312, J7637, J7638, J1700, J1710, J1720, J1020, J2030, J1040, J2920, J2930, J7509, J2650, J7510, J7506, J7512, J3300, J3301, J3302, J3303, J7683, J7684
Diuretics	37%, 369918%, 369920%, 369940%, 369945%, 369950%, 369955%, 369960%, 369968%, 369990%	J1120, S0171, J1940, J3265, J2150, J7665, J3350, J1205,

Abbreviations: GPI, generic product identifier; HCPCS, Healthcare Common Procedural Coding System; N/A, not applicable.

 © 2022. The Author(s).

Supplemental Table S5 Codes for medication relevant to the management of coagulopathy

Medication class	Subclass, if applicable	Drug name	Routes of administration	GPI code	HCPCS code	ICD-10-CM procedure
Anticoagulant	Vitamin K antagonist	Warfarin	Oral	83200030%		
	Factor Xa inhibitor	Apixaban	Oral	83370010%		
		Rivaroxaban	Oral	83370060%		
		Edoxaban	Oral	83370030%		
		Betrixaban	Oral	83370018%		
	Unfractionated heparin (UFH)	Heparin	IV	83100020%	J1642, J1644	
	Low molecular weight heparin (LMWH)	Dalteparin	SQ	83101010%	J1645	
		Enoxaparin	SQ	83101020%	J1650	
		Fondaparinux	SQ	83103030%	J1652	
	Direct thrombin inhibitor (DTI)	Dabigatran	Oral	83337030%		
		Lepirudin	IV	83334050%	J1945	
		Desirudin	IV	83334030%		
		Bivalirudin	IV	83334020%	J0583	
		Argatroban	IV	83337015%	J0883, J0884	
Antiplatelet		Aspirin	Oral	64100010%		
		Clopidogrel	Oral	85158020%		
		Prasugrel	Oral	85158060%		
		Ticlodipine	Oral	85158080%		
		Dipyridamole	Oral	85150030%		
		Ticagrelor	Oral	85158470%		
		Cangrelor	IV	8515842520%		
		Aspirin and dipyridamole	Oral	85159902206920		
		Cilostazol	Oral	85155516%		
Tissue plasminogen activator		Alteplase	IV	85601010%	J2997	
		Anistreplase	IV	85601020%	J0350	
		Reteplase	IV	85601070%	J2993	
		Tenecteplase	IV	85601075%	J3101	
Antivirals		Remdesivir	IV	12700080%		XW033E5, XW043E5
Corticosteroids		Dexamethasone	Oral, IV	22100020%	J1094, J1100, J8540	
		Prednisone	Oral, IV	22100045%	J7506, J7512	
		Methylprednisolone	Oral, IV	22100030%	J1020, J1030, J1040, J2920, J2930, J7509	
		Hydrocortisone	Oral, IV	22100025%	J1700, J1710, J1720	
Convalescent plasma						XW13325, XW14325
Antithrombin III		Atryn	IV	85400015202120		
		Thrombate III	IV	854000151021%		

Abbreviations: GPI, generic product identifier; HCPCS, Healthcare Common Procedural Coding System; ICD-10-CM, *International Classification of Diseases, 10th Revision, Clinical Modification*; IV, intravenous; SQ, subcutaneous.

 © 2022. The Author(s).

Supplemental Table S6 Codes for thromboembolic events of interest

Thromboembolic event	ICD-10-CM diagnosis/procedure codes	CPT codes
Ischemic stroke	I63.0%	N/A
Myocardial infarction	I21.0%, I22.0%	N/A
Deep vein thrombosis	I80.1%, I80.2%, I80.3%, I82.4%, I82.6%, I82.A1%, I82.B1%, I82.C1%, I82.90%	N/A
Pulmonary embolism	I26.0%	N/A
Acute limb ischemia	I70.22, I70.25, I70.26	N/A
Major nontraumatic lower limb amputation	0Y62, 0Y63, 0Y64, 0Y67, 0Y68, 0Y6C, 0Y6D, 0Y6F, 0Y6G, 0Y6H, 0Y6J, 0Y6M, 0Y6N, 0Y6P, 0Y6Q, 0Y6R, 0Y6S, 0Y6T, 0Y6U, 0Y6V, 0Y6W, 0Y6X, 0Y6Y	27290, 27295, 27590, 27591, 27592, 27594, 27596, 27598, 27880, 27881, 27882, 27884, 27886, 27888, 27889, 28130, 28140, 28800, 28805, 28810, 28820, 28825, 28124, 28126, 28150, 28153, 28160

Abbreviations: CPT, current procedural terminology; ICD-10-CM, *International Classification of Diseases, 10th Revision, Clinical Modification*; N/A, not applicable.

Supplemental Table S7 Demographics and baseline[a] clinical characteristics of overall COVID-19 cohort

Number of patients	181,995
Age, years	
Mean (SD)	49.99 (18.70)
Age group, years, *n* (%)	
18–24	18,047 (9.92)
25–34	27,893 (15.33)
35–44	28,217 (15.50)
45–54	31,565 (17.34)
55–64	33,661 (18.50)
65+	42,612 (23.41)
Sex, *n* (%)	
Male	81,325 (44.69)
Female	100,670 (55.31)
Race, *n* (%)	
African American	23,752 (13.05)
Asian	3,366 (1.85)
Caucasian	128,741 (70.74)
Other/unknown	26,136 (14.36)
Ethnicity, *n* (%)	
Hispanic	20,938 (11.50)
Not Hispanic	145,367 (79.87)
Unknown	15,690 (8.62)
Geographic region, *n* (%)	
Northeast	28,874 (15.87)
West	11,807 (6.49)
Midwest	99,124 (54.47)
South	35,767 (19.65)
Other/unknown	6,423 (3.53)

 © 2022. The Author(s).

Supplemental Table S7 (*Continued*)

Number of patients	181,995
Insurance type, *n* (%)	
Commercial	83,398 (45.82)
Medicaid	12,062 (6.63)
Medicare	18,710 (10.28)
Others	35,878 (19.71)
Uninsured	2,196 (1.21)
Unknown	4,021 (2.21)
Missing	25,730 (14.14)
BMI (kg/m^2)	
Patients with BMI measure, *n* (%)	113,991 (62.63)
Mean BMI (SD)[b]	31.52 (8.08)
BMI category, *n* (%)[b]	
Underweight	1,091 (0.96)
Normal	21,271 (18.66)
Overweight	32,486 (28.50)
Obese	59,143 (51.88)
Obesity class, *n* (%)[b]	
Class 1 (BMI 30.0–34.9 kg/m^2)	27,592 (24.21)
Class 2 (BMI 35.0–39.9 kg/m^2)	16,541 (14.51)
Class 3 (BMI $\geq$40 kg/m^2)	15,010 (13.17)
QCI, mean (SD)	0.62 (1.39)
Individual comorbidity, *n* (%)	
Hypertension	52,419 (28.80)
Hyperlipidemia	44,210 (24.29)
Diabetes	26,001 (14.29)
Anxiety	22,540 (12.38)
Depression	18,466 (10.15)
Thyroid disease	16,832 (9.25)
Osteoarthritis	15,289 (8.40)
Anemia	14,218 (7.81)
Asthma	12,027 (6.61)
Chronic kidney disease	11,185 (6.15)
Sleep apnea	10,713 (5.89)
Nonalcoholic fatty liver disease	10,307 (5.66)
Chronic obstructive pulmonary disease	7,629 (4.19)
Congestive heart failure	7,493 (4.12)
Cancer[c]	7,381 (4.06)
Prior stroke/transient ischemic attack	4,698 (2.58)
Osteoporosis	3,670 (2.02)
Peripheral vascular disease	3,634 (2.00)
Old MI	3,109 (1.71)
Rheumatoid arthritis	1,960 (1.08)
Liver cirrhosis	1,033 (0.57)

(*Continued*)

 © 2022. The Author(s).

Supplemental Table S7 (*Continued*)

Number of patients	181,995
Stable angina	1,044 (0.57)
Unstable angina	432 (0.24)
Uveitis	136 (0.07)
Prior thromboembolic event, *n* (%)	
Ischemic stroke	2,118 (1.16)
MI	1,990 (1.09)
DVT	1,471 (0.81)
PE	955 (0.52)
ALI	183 (0.10)
Major nontraumatic lower limb amputation	272 (0.15)

Abbreviations: ALI, acute limb ischemia; BMI, body mass index; COVID-19, coronavirus 2019; DVT, deep vein thrombosis; MI, myocardial infarction; PE, pulmonary embolism; QCI, Quan–Charlson comorbidity index; SD, standard deviation.
[a]The baseline period was defined as the 12 months prior to the index date.
[b]Mean BMI, BMI categories, and obesity classes were identified among hospitalized patients with a BMI measure available ($n = 113,991$).
[c]A diagnosis for cancer required two diagnosis codes for the same type of cancer at least 30 days apart.

Supplemental Table S8 Exploratory analysis: feasibility of assessing risk stratification scores in hospitalized COVID-19 patients ($n = 40,524$)

Score	Component	Patients with value, *n* (%)
NOCOS calculator https://feinstein.northwell.edu/NOCOS	Age	40,524 (100.00)
	Oxygen saturation (%)	39,015 (96.28)
	Absolute neutrophil count (K/uL)	39,223 (96.79)
	Red cell distribution width (%)	40,299 (99.44)
	Serum sodium (mmol/L)	26,645 (65.75)
	Serum blood urea nitrogen (mg/dL)	39,591 (97.70)
IMPROVE-DD VTE risk score The IMPROVE-DD VTE risk assessment algorithms (practical-haemostasis.com)	Previous VTE	1,108 (2.73)
	Known thrombophilia	427 (1.05)
	Current lower limb paralysis	110 (0.27)
	Current cancer	2,181 (5.38)
	Immobilized $\geq$7 d	24,579 (60.65)
	ICU or CCU stay	8,630 (21.30)
	Patient age >60 y	24,501 (60.46)
	D-dimer level[a]	30,153 (74.41)

Abbreviations: CCU, cardiac care unit; COVID-19, coronavirus 2019; ICU, intensive care unit; NOCOS, Northwell COVID-19 Survival; VTE, venous thromboembolism.
[a]Patients with available D-dimer level. Note the risk score cutoff is two times the upper limit of normal.

 © 2022. The Author(s).

Supplemental Table S9 Subgroup analysis: thromboembolic events during follow-up period by ICU stay[a]

	Hospitalized COVID-19 patients ($n = 40{,}524$)	
	**Without ICU stay**	**With ICU stay**
Number of patients	31,894	8,630
Occurrence of thromboembolic events, *n* (%)		
Any of the thromboembolic events of interest[b]	5,030 (15.77)	2,083 (24.14)
Ischemic stroke	1,122 (3.52)	422 (4.89)
MI	2,219 (6.96)	900 (10.43)
DVT	1,136 (3.56)	625 (7.24)
PE	1,307 (4.10)	542 (6.28)
ALI	71 (0.22)	18 (0.21)
Major nontraumatic lower limb amputation	96 (0.30)	22 (0.25)
Time to first event (days), mean (SD)[c]		
Any of the thromboembolic events of interest[b]	16.2 (36.9)	12.1 (26.3)
Ischemic stroke	25.8 (50.3)	18.1 (36.9)
MI	13.7 (34.3)	8.8 (21.7)
DVT	24.4 (45.0)	18.6 (32.9)
PE	15.3 (32.4)	12.9 (23.4)
ALI	44.0 (60.2)	18.1 (43.5)
Major nontraumatic lower limb amputation	33.6 (58.0)	33.3 (47.4)

Abbreviations: ALI, acute limb ischemia; COVID-19, coronavirus 2019; DVT, deep vein thrombosis; ICU, intensive care unit; MI, myocardial infarction; PE, pulmonary embolism; SD, standard deviation.
[a]ICU stay was identified during COVID-19 hospitalization.
[b]The thromboembolic events of interest included ischemic stroke, MI, DVT, PE, ALI, and major nontraumatic lower limb amputation.
[c]Time to first event was examined among patients with the event.

 © 2022. The Author(s).
